# Pattern-Based Sinkhole Detection in Arid Zones Using Open Satellite Imagery: A Case Study Within Kazakhstan in 2023

**DOI:** 10.3390/s25030798

**Published:** 2025-01-28

**Authors:** Simone Aigner, Sarah Hauser, Andreas Schmitt

**Affiliations:** 1 Geoinformatics Department, Hochschule München University of Applied Sciences, Karlstraße 6, D-80333 Munich, Germany; 2 Institute for Applications of Machine Learning and Intelligent Systems, Lothstraße 34, D-80335 Munich, Germany

**Keywords:** remote sensing, Sentinel-2, Sentinel-1, hyper-complex bases, sinkholes, relief, digital elevation model, multi-scale filter banks

## Abstract

Sinkholes are significant geohazards in karst regions that pose risks to landscapes and infrastructure by disrupting geological stability. Usually, sinkholes are mapped by field surveys, which is very cost-intensive with regard to vast coverages. One possible solution to derive sinkholes without entering the area is the use of high-resolution digital terrain models, which are also expensive with respect to remote areas. Therefore, this study focusses on the mapping of sinkholes in arid regions from open-access remote sensing data. The case study involves data from the Sentinel missions over the Mangystau region in Kazakhstan provided by the European Space Agency free of cost. The core of the technique is a multi-scale curvature filter bank that highlights sinkholes (and takyrs) by their very special illumination pattern in Sentinel-2 images. Marginal confusions with vegetation shadows are excluded by consulting the newly developed Combined Vegetation Doline Index based on Sentinel-1 and Sentinel-2. The geospatial analysis reveals distinct spatial correlations among sinkholes, takyrs, vegetation, and possible surface discharge. The generic and, therefore, transferable approach reached an accuracy of 92%. However, extensive reference data or comparable methods are not currently available.

## 1. Introduction

Modern infrastructure requires a stable base. While the stability of the subsoil in densely populated areas is well mapped, hardly anything is known about new remote areas to be developed, for example, for the generation of green energy, as is the case in Kazakhstan.

### 1.1. Problem Statement and Relevance

Sinkholes, also known as dolines, are prevalent in various global environments, ranging from arid to humid regions, and their formation is influenced by diverse geological and environmental factors [1]. Sinkholes occur in areas where rocks consist of calcium carbonates, such as dolomite and limestone. These rocks, which dissolve easily in water, are known as karst. Sinkholes form naturally when groundwater dissolves these geological units and forms underground karst cavities over time. The cavities grow towards the surface until the overlying structure collapses. They are usually circular and have a diameter of several dozen metres to over a kilometre and a depth of several metres to hundreds. Their shape varies from shallow basins to steep-walled cylindrical forms depending on the formation process [2].

Sinkholes pose a variety of risks that extend beyond their geophysical characteristics. These geological phenomena pose a major hazard, especially in karst regions where sudden ground collapses can cause major damage including severe economic loss (such as extensive damage to infrastructure like roads, bridges, or buildings), injuries, and human life loss [3,4,5]. These incidents, although not always at such extremes, emphasise the unpredictable and potentially dangerous nature of sinkholes, which are difficult to detect and accurately assess [6]. In addition, sinkholes pose ecological risks due to the direct connection between surface water and groundwater. As a result, pollutants from agricultural chemicals, industrial waste, or wastewater can enter the groundwater, which can affect drinking water quality and, consequently, public health [7]. The dual role of sinkholes as both geohazards and ecological vulnerabilities underscores the need for advanced detection and monitoring strategies. Robust detection, monitoring, and prevention methods are essential for the effective risk management of sinkholes. Automated sinkhole detection could improve risk assessment, support planning, and help to reduce risks to the environment and human safety. This research seeks to address these challenges through the development of innovative methods that leverage freely available data for scalable and, at the same time, cost-effective sinkhole detection.

### 1.2. State of the Art

The identification and mapping of sinkholes, or dolines, have been explored extensively through a variety of methodologies, primarily categorised into geophysical methods, geographic information system (GIS)-based analyses, remote sensing techniques, and hybrid approaches. Each approach has unique advantages and limitations, influencing its applicability for sinkhole detection at different scales and in varying environmental conditions.

Field surveys and ground-based approaches play a fundamental role in the identification and mapping of sinkholes, offering detailed insights into both surface and subsurface characteristics. One of the simplest methods is visual inspection, where surface features like depressions, cracks, and instability signs are manually mapped. Although widely used, this method is time-consuming, subjective, and less effective in vegetated or urbanised areas. More advanced are speleological explorations, which involve examining caves and karst voids to identify structures like collapse chimneys or sediment-filled pipes, aiding in predicting future sinkhole activity [8,9,10].

A variety of geophysical techniques provide non-invasive insights into subsurface anomalies. Electrical resistivity imaging (ERI) detects subsurface conductivity changes to identify voids or water-filled cavities [11], while ground-penetrating radar (GPR) uses electromagnetic waves to map near-surface density variations, particularly effective for shallow karst systems [12,13]. Gravimetry and magnetometry extend these capabilities by identifying larger-scale subsurface anomalies, which are valuable for detecting extensive sinkhole clusters [14,15]. For direct subsurface information, drilling and probing techniques are commonly employed. Boreholes reveal geotechnical properties, such as voids and disturbed sediments, and hydrogeological data like water table fluctuations [16]. However, these methods are resource-intensive and risk missing larger cavities due to limited borehole coverage. Complementary to drilling, trenching involves excavating across suspected sinkholes to determine their boundaries, mechanisms, and formation history [11,17].

GIS-based analysis has revolutionised sinkhole detection and mapping by enabling efficient and large-scale spatial analysis of karst landscapes. One key tool is the use of topographic maps, which help identify sinkholes based on terrain depressions and contours. However, the accuracy depends on the map’s resolution and scale, as smaller sinkholes may be overlooked [18,19]. Historical topographic maps can also reveal sinkholes that are now covered by vegetation or urban development, providing valuable insights into past karst activity [20,21]. Although GIS-based methods are powerful, their effectiveness is often limited by the availability and resolution of input data. Additionally, manual interpretation in GIS environments can be time-intensive and prone to human error. Despite these challenges, GIS remains a cornerstone in sinkhole studies due to its capability to integrate diverse datasets and perform sophisticated spatial analyses.

Remote sensing techniques have become indispensable for detecting and analysing sinkholes, particularly for large-scale or inaccessible areas. These methods rely on data captured from satellites, aircraft, or drones to identify surface features and subsidence patterns indicative of sinkhole activity. Optical imagery is one of the most widely used remote sensing tools, where satellite data such as Landsat [22], RapidEye [23], and IKONOS [24] are analysed to detect surface anomalies. GIS platforms also integrate remote sensing imagery with morphometric parameters to analyse the spatial patterns of sinkholes over time. Historical aerial images and satellite data such as Landsat and Sentinel-2 data have been used to track the evolution and frequency of sinkhole formation, correlating these trends with environmental or anthropogenic factors [25,26]. In northwest Morocco, for instance, regions prone to karstification were identified by deriving vegetation and water indices from satellite images, highlighting areas with higher surface water input [27]. Similarly, aerial photographs combined with orthorectified images and GIS platforms are used to identify historical or masked sinkholes and assess their temporal evolution [25,28]. Digital tools such as digital elevation models (DEMs) are extensively used to analyse the topography and morphology of sinkholes. High-resolution DEMs derived from LiDAR (light detection and ranging) or satellite imagery allow researchers to automatically detect and characterise sinkholes based on geometric parameters like depth, perimeter, and slope [29]. LiDAR provides high-resolution topographic data, enabling the detection of sinkholes and associated features even in vegetated or remote areas. LiDAR-derived DEMs allow for the automatic mapping of sinkholes by analysing their geometric properties, such as depth and diameter. This method is particularly effective for morphometric characterisation and detecting subtle subsidence patterns that may precede sinkhole formation [30,31]. Furthermore, LiDAR has been successfully applied in Slovenia, where high-resolution data increased sinkhole detection accuracy to 83.5% [29]. Aerial photographs, combined with DEMs, help validate and refine sinkhole mapping by visually confirming subsidence features and vegetation changes [32]. Furthermore, swath bathymetry, a specific form of DEM analysis, is applied in underwater environments to detect sinkholes on lakebeds or ocean floors [33]. Techniques like automatic mapping and photogrammetry further enhance detection accuracy, enabling efficient sinkhole monitoring over large areas [34]. Radar-based techniques, such as InSAR (interferometric synthetic aperture radar), are used to monitor ground deformation over large areas. Differential interferometry (DInSAR) is particularly effective for measuring subsidence rates and temporal changes in karst regions. In the Ebro Valley, Spain, DInSAR velocity maps were cross-referenced with sinkhole inventories to assess doline activity and predict future collapses [35]. Although radar methods excel at capturing broad subsidence patterns, small active sinkholes or rapid collapses may be overlooked due to their spatial resolution limitations. Recent advancements, such as the Sinkhole Scanner method [36], address some of these limitations by employing a two-dimensional Gaussian function to detect sinkhole-related spatio-temporal patterns in InSAR deformation time series. This method, tested on Sentinel-1A data, successfully detected sinkholes even in challenging environments, demonstrating stability in arid regions and improved detection in vegetated areas, a key advantage for addressing the dual challenges of sinkhole monitoring in such conditions. However, the Sinkhole Scanner is not without limitations. Its reliance on a Gaussian kernel assumes specific deformation shapes, potentially missing irregular or complex sinkhole patterns. Furthermore, the method may struggle to detect very rapid collapses without precursory signals, and its computational intensity can be a limiting factor when analysing large, high-resolution datasets. While it improves detection in vegetated areas, challenges like signal decorrelation persist, especially in regions with dense vegetation. Multispectral and hyperspectral imaging further enhance sinkhole detection by revealing vegetation stress, soil moisture changes, or water pooling—indirect indicators of subsurface karst processes. Airborne multispectral scanning, for example, has shown promise in detecting subtle environmental changes linked to sinkhole formation [37]. These remote sensing techniques are often complemented by manual processing in GIS environments or field verification to confirm suspected sinkholes. However, their reliance on high-resolution datasets and specialised software can make them resource-intensive and less accessible for widespread or continuous monitoring. Despite these challenges, remote sensing remains a cornerstone in modern sinkhole research, particularly for large-scale mapping and long-term monitoring.

In order to be accessible for all potential users, the data should be open. Additionally, to guarantee large coverage, the data have to be globally available. Preceding studies have shown that optical data are most promising. Three satellite missions fulfill these requirements: Sentinel-2 [38], Landsat [22], and MODIS [39]. According to the literature, the spatial resolution is playing a key role in the detection of sinkholes, whereas the temporal resolution is negligible. In this sense, Sentinel-2 (10 m pixels every 5 days) outperforms existing open sources such as Landsat (30 m every 16 days) and MODIS (250 m every 1–2 days).

Artificial intelligence (AI) is increasingly being applied to sinkhole detection and mapping, offering a new paradigm for automating complex analyses and enhancing accuracy. Machine learning (ML) and deep learning techniques, in particular, have shown significant promise by processing large datasets, identifying patterns, and predicting sinkhole-prone areas with minimal human intervention. One of the most notable applications of AI is in image recognition and object detection. For example, the YOLO (you only look once) algorithm has been used to detect sinkholes in satellite and aerial imagery with handsome results. In a study conducted in Kazakhstan, YOLO achieved a detection accuracy of 74% for sinkholes and 86% for geological pre-sinkhole features such as takyr depressions [40]. Also, a sinkhole-tracking methodology that employed CNN transfer learning on FIR imagery was successfully implemented [41]. These methods enable rapid, large-scale detection, outperforming traditional manual and semi-automated techniques in both speed and efficiency. Supervised learning algorithms have been employed to classify land features and identify potential sinkhole zones. These models are trained using labelled datasets of known sinkholes and environmental variables such as topography, hydrology, and geology. Once trained, the models can predict high-risk areas by analysing similar patterns in new datasets. While highly accurate, this approach requires comprehensive and reliable training data, which can be difficult to obtain for regions with sparse sinkhole documentation. Unsupervised learning and clustering methods are other emerging avenues. These techniques analyse unlabelled data to identify anomalies or clusters indicative of sinkhole-prone regions. They are particularly useful for preliminary assessments in regions where ground truth data are limited. AI also plays a critical role in temporal analysis, helping to track sinkhole evolution and predict collapses. Time-series data from remote sensing platforms, such as LiDAR or InSAR, can be fed into recurrent neural networks (RNNs) or long short-term memory (LSTM) models to detect subtle deformation trends and assess the likelihood of future events. Despite these advances, challenges remain in the widespread application of AI to sinkhole studies. The creation of robust and diverse training datasets is often labour-intensive, and the computational demands of deep learning models can be prohibitive for smaller research teams or institutions. Additionally, the interpretability of AI models is sometimes limited, making it difficult to understand the underlying decision-making process and validate the results.

### 1.3. Challenges

Detecting sinkholes poses significant challenges with respect to low data availability, spatial resolution constraints, and large extents. The problem is becoming topical as there are plans to develop former unused land in places such as the Mangystau area in Kazakhstan. Research on sinkhole formation mechanisms and hazards in Kazakhstan remains sparse [42,43], and the precise delineation of sinkholes at finer scales continues to be an unsolved topic [44]. Digital terrain models (DTMs), especially those derived from LiDAR [29,45], are widely regarded as the optimal data source for sinkhole detection. These models provide high-resolution representations of the bare-earth surface, crucial for identifying the subtle depressions characteristic of sinkholes. However, high-resolution DTMs are often unavailable, even in regions with advanced geospatial infrastructure. For instance, in Bavaria, DTMs with 1 m resolution are typically limited in temporal and geographic coverage [46]. This lack of temporal consistency hinders long-term monitoring and dynamic geohazard assessment. In Kazakhstan, the absence of high-resolution DTMs presents a significant barrier to applying conventional detection methodologies. Kazakhstan’s geographic scale amplifies these challenges. Covering over 2.7 million square kilometers, it is among the largest countries globally, but its low population density makes large-scale mapping and monitoring logistically and economically prohibitive. Even smaller, resource-rich regions like Bavaria struggle to maintain consistent, high-quality geospatial data, illustrating the difficulty of conducting geomorphological studies at Kazakhstan’s scale. In the absence of DTMs, digital elevation models are often used as substitutes [44]. However, unlike DTMs, DEMs include surface features such as vegetation and structures, which can obscure the underlying terrain and limit their effectiveness for detecting small-scale geomorphic features. While DEMs can capture larger deformations, their typically coarse resolution (e.g., 30 m in widely available datasets like the Copernicus DEM) restricts their utility for identifying subtle or small sinkholes. Additionally, inconsistent spatial and temporal availability further reduces their effectiveness for large-scale or continuous geohazard monitoring. The lack of high-resolution DTMs and the limitations of DEMs highlight a critical research gap in Kazakhstan. Traditional sinkhole detection approaches, reliant on detailed terrain data, are poorly suited to the region’s data-sparse context. These challenges require innovative and cost-effective methods that utilise widely available resources such as freely accessible remote sensing imagery. By exploiting shadow effects and natural illumination in these datasets, it is possible to enhance the visibility of geomorphic depressions, even in the absence of high-resolution terrain models. Such methods are particularly well-suited to Kazakhstan’s sparsely vegetated landscapes, where minimal surface obstructions favour the use of optical data. Developing a scalable, automated method based on remote sensing not only mitigates the limitations of existing datasets but also offers a practical and adaptable solution for geohazard assessments in large, under-explored regions like Kazakhstan. By addressing the current data gaps and harnessing the potential of remote sensing technologies, this study aims to provide a novel framework for sinkhole detection that is both accessible and applicable across similar geographic contexts.

### 1.4. Research Hypotheses

The novel approach addresses the shortcomings of conventional methods in terms of coverage, the use of freely available data sources, and operationalisability. The reliability of the results is one major aspect of this study. Finally, the geospatial analysis of the results will lead to new insights to the geology. One can summarise the goal in three research hypotheses that will guide the reader through the article.

1. Natural illumination by the sun can highlight terrain features that support the automated identification of sinkholes by multi-scale filtering approaches from image processing.
2. The automatically derived results are plausible and even allow for the discrimination of sinkholes from other morphologically similar terrain features like takyrs and their surrounding vegetation.
3. There are clear spatial correlations between the occurrence of sinkholes and the visible or modelled surface discharge by superficial water bodies estimated by the help of a DEM-based accumulation model.

The following sections introduce the study site and the data. The methodology is briefly described and the results are illustrated. The discussion and the conclusion close the article.

## 2. Study Site

**Figure 1:**
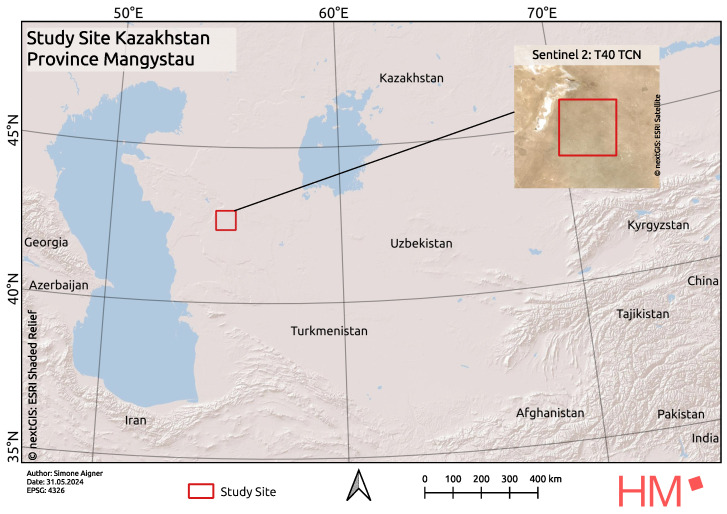
The study site is located in the province of Mangystau, Kazakhstan. A red rectangle highlights the area of interest; a detailed view in the form of a Sentinel-2 image can be seen in the enlargement.

The selected study area is located in southwestern Kazakhstan, within the southeastern part of the Mangystau Province, near the Caspian Sea (Figure 1).

The Ustyurt Plateau, covering approximately 5000 square kilometers near the borders of Turkmenistan and Uzbekistan, is adjacent to the Ustyurt National Reserve but falls outside the protected area. This region’s sparse development and lack of significant human settlement, evidenced by minimal infrastructure and occasional pathways observed via satellite imagery [47,48], underline its status as a largely untouched and unexplored landscape. Geological investigations are crucial here to address potential challenges and opportunities presented by the area’s complex subsurface features. This need has become even more pressing in light of the increasing interest in developing large-scale renewable energy projects in southwestern Kazakhstan. These initiatives aim to harness the region’s abundant wind and solar resources to generate green hydrogen through water electrolysis, which can then be converted into ammonia for efficient storage and transportation. However, the presence of sinkholes in the proposed development areas poses a notable geological challenge, potentially impacting the stability and sustainability of wind and solar installations. To address these risks, detailed geological assessments and continuous monitoring are essential. Identifying and mitigating the impacts of karst structures can support the safe and effective implementation of renewable energy infrastructure. Automated detection and monitoring systems play a critical role in reducing risks, informing planning processes, and enhancing the long-term feasibility of such projects.

**Figure 2:**
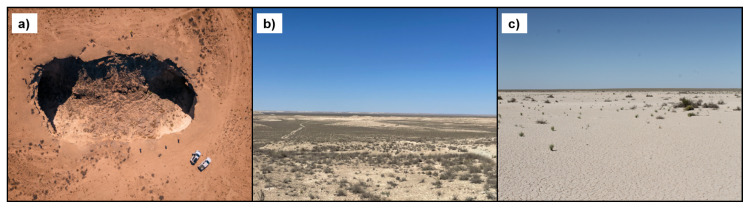
(**a**) Drone-captured aerial view of a sinkhole, showcasing its circular morphology; (**b**) the broader karst/desert region featuring characteristic shrub and grass vegetation; (**c**) typical flat terrain (takyrs) common to the region [49].

The geomorphology of Mangystau is dominated by karst processes, erosion, and tectonic activity. This arid region features unique structures such as steep depressions and sinkholes formed through the dissolution of carbonate rocks. Figure 2, adapted from data provided by Svevind Energy Group [49], illustrates key geomorphological features of the study area, including a drone-captured aerial view of a sinkhole showcasing its circular morphology, the broader karst and desert landscape with sparse vegetation, and takyr plains, which are flat, cracked clay surfaces characteristic of arid regions. These features highlight the suitability of the area for remote sensing applications, particularly sinkhole detection and spatial analysis. The prevalent soil type in the study area is Calcisol, which is typical of arid environments and characterised by high calcium carbonate content near the surface. These soils often support only sparse vegetation, such as drought-resistant shrubs and grasses, and exhibit circular vegetation patterns that correspond to subsurface karst structures, including sinkholes [50]. In addition, the region contains takyr plains, which are clay-rich surfaces with polygonal cracking patterns. These plains reflect a history of marine sedimentation, often bearing salt deposits, and their unique surface texture makes them important for understanding hydrological processes and subsurface stability [51]. The climate in Mangystau is arid and desert-like, with significant contrasts between humid and arid seasons. Precipitation is rare and irregular, and temperatures show dramatic seasonal variation. Winters are relatively mild due to the moderating influence of the Caspian Sea, while summers are extremely hot and arid, often accompanied by strong winds that drive erosion and sediment redistribution [52]. The sparse vegetation in the area is dominated by shrubs and grasses [53,54], with more robust growth near depressions or along the edges of takyr plains, where water may temporarily accumulate. These climatic and vegetative conditions are significant for understanding surface and subsurface processes in the context of sinkhole detection.

The region’s geological history is also reflected in its soils and landforms. Marine sedimentation in ancient times [55] contributed to the development of takyr plains and evaporitic deposits, highlighting the area’s evolution from a submerged marine environment to its current arid state. This history, combined with the area’s unique karst features, provides a critical context for studying subsurface vulnerabilities and developing sustainable land-use strategies.

## 3. Data

In the study site of Kazakhstan, sinkhole detection relies on satellite image data from multiple sources, enabling a robust analysis of geological features. Freely available DEM data play a role in the spatial analysis. In addition, different reference data are used for validation.

### 3.1. Synthetic Aperture Radar

Sentinel-1, part of the European Space Agency’s (ESA) Copernicus program, data were employed to provide synthetic aperture radar (SAR) data for this study. Specifically, data from the Sentinel-1A (©ESA) satellite were used, acquired in Interferometric Wide Swath (IW) mode. This mode delivers high-resolution Single Look Complex (SLC) data, preserving amplitude and phase information for advanced analyses. The data were collected on 6 August 2023, using VV+VH dual-polarisation. This configuration provides detailed backscatter information, making it suitable for analysing surface properties such as vegetation structure and soil moisture. Sentinel-1’s SAR capabilities enable reliable monitoring under all weather and lighting conditions, including cloud cover and nighttime, complementing the optical dataset used in this study.

### 3.2. Multispectral Images

ESA’s Sentinel-2 data provide high-resolution multispectral imagery with a spatial sampling of 10 m for the key spectral bands (blue, green, red, and near-infrared), enabling advanced image processing techniques and index development for geological and vegetation analysis. Its polar orbit and sun-synchronous orientation ensure consistent image acquisition, essential for monitoring seasonal and surface changes under arid and vegetated conditions. Sentinel-2 Level-2A (©ESA) data deliver Bottom of Atmosphere (BOA) reflectance values, enhancing the quality and reliability of the analyses. Table 1 summarises the used Sentinel-2 data with their respective cloud coverage from October 2022 to September 2023.

### 3.3. Digital Elevation Model

The digital elevation model (DEM) used in this study is the Copernicus DEM, which provides freely available surface data at a resolution of 30 m. As a surface model, it represents the Earth’s topography, including vegetation, buildings, and infrastructure, and is based on the WorldDEM dataset [56]. Its spatial resolution is much too coarse to detect sinkholes, but it is was applied in this study to derive contour lines as well as to perform a water flow simulation with the SAGA tool in QGIS [57]. The flow accumulation is calculated using the multiple flow direction method according to Freeman [58]. This analysis is critical for understanding the spatial distribution of water flow, which has implications for watershed delineation and its relationship to karst features in the region. Additionally, the DEM provides essential elevation data that support the spatial analysis of geomorphological structures. While the sampling of 30 m is sufficient for large-scale analyses, it poses limitations for the direct detection of small sinkholes, as features smaller than about 100 m cannot sufficiently be resolved. However, in the Mangystau study area, where vegetation is sparse but characterised by fine-scale patterns, the DEM is still effective for deriving surface flow patterns and broader topographic insights. These insights are particularly valuable for assessing hydrological and geomorphological processes, even when smaller sinkholes or vegetation features are below the DEM’s resolution.

### 3.4. Reference Data for Sinkholes, Takyrs, and Vegetation

To validate the identification of sinkholes, reference data were provided by Svevind Energy Group [49]. This ground-truth dataset includes a shapefile containing GNSS-based measurements of 13 known sinkholes, collected on-site in 2023 using handheld GNSS equipment. These measurements were performed by a regional topographer to provide precise geospatial locations for these features. In addition to the GNSS data, georeferenced imagery of the sinkholes [49], also captured in 2023, is available. These combined datasets represent the confirmed ground-truth information for sinkholes in the study area and serve as primary references for the analysis.

The Soviet military maps integrated into the Atlogis Topomapper [48] tool proved valuable for validating the takyr detection. These detailed and accurate maps, created during the Cold War, were partially published after 1991 and remain an important source for regions with limited current data [59]. The marked takyrs and their surrounding vegetation were digitised as reference features.

High-resolution World Imagery [60] was utilised to analyse vegetative patterns across the study area. This dataset, provided by Esri, offers detailed satellite and aerial imagery with a spatial resolution ranging from 0.3 m to 15 m globally, depending on the source and location. The imagery includes contributions from multiple commercial and governmental sources, seamlessly integrated into a global mosaic. Vegetative features such as dense and sparse vegetation were visually derived from this dataset, allowing for the precise identification and delineation of vegetation classes that are difficult to map in remote or semi-arid environments. The high level of spatial detail provided by World Imagery enabled the analysis of fine-scale vegetative patterns across the study area, serving as a critical reference for understanding land cover dynamics in southern Kazakhstan.

The Proba-V LC100 global land cover product from 2019 [61] provided another key dataset for analysing vegetation. This dataset is part of the Copernicus Global Land Service and was derived from observations collected by the Proba-V satellite, launched by the ESA in 2013. Proba-V operates with a spatial resolution of 100 m for vegetation mapping and collects data using a wide field-of-view imaging spectrometer across the visible and near-infrared spectrum. The LC100 product provides fractional cover values for grass and shrub vegetation, expressed as percentages from 0–100%, and employs advanced algorithms to classify land cover types consistently across the globe. These fractional values are generated through a combination of vegetation indices and supervised classification techniques. With its reliable precision and global consistency, Proba-V offers a robust dataset for regional-scale vegetation analysis, enabling the detailed characterisation of grass and shrubland distribution in semi-arid landscapes such as southern Kazakhstan.

## 4. Methodology

The method is made up of several procedural work stepswhich are linked together and converge to form a final overall analysis.

### 4.1. Preparation of Image Data

To detect and extract prominent features, such as sinkholes and takyrs, a systematic approach to imagery preprocessing was undertaken. Sentinel-2 data were processed into six distinct datasets, each designed to enhance the analysis of temporal and spectral variations under varying environmental conditions. These datasets capture both long-term stability and seasonal contrasts, facilitating the effective detection of features in arid and semi-arid landscapes. The preprocessing steps included the calculation of total intensity using Kennaugh elements [62], spectral analysis using principal component analysis (PCA), and the creation of a difference image to emphasise seasonal contrasts. Table 2 summarises the datasets and their specific purposes.

The two main approaches were the calculation of total intensity using Kennaugh elements (unweighted) and the weighted analysis of spectral variables using principal component analysis (PCA). To capture seasonal variations, February (humid conditions) and August (arid conditions) were selected as representative months. February reflects increased precipitation and vegetation cover due to lower temperatures, while August is characterised by higher temperatures, lower vegetation, and drier soil conditions. In addition, the data from all 12 months were combined into a stable temporal-spectral dataset by multi-temporal fusion. A difference image between February and August was also created to maximise seasonal contrasts and improve feature identification. This pre-processing resulted in six datasets.

The first dataset processed was the temporal-spectral dataset, which was created by averaging the spectral and temporal information over the 12 months to account for long-term stability. The second and third datasets represented the overall intensity based on Kennaugh elements for February (humid conditions) and August (arid conditions), respectively. The Kennaugh approach, derived from SAR polarimetry, is a technique in which optical bands are transformed using quaternion bases [63]. For the Sentinel-2 optical data (B2 = Blue, B3 = Green, B4 = Red, and B8 = NIR), this method was adapted within a unified radiometric framework known as Hypercomplex Bases (HCB) [63]. The Sentinel-2 reflectance values are normalised to reduce the dominance of the NIR band and ensure spectral consistency. These normalised values are then converted into Kennaugh-like elements, including a total reflectance element ($K_{0}$) and three spectral elements. In this case, the spectral Kennaugh-like element $K_{0}$ was used as an unweighted sum of the spectral bands (blue, green, red, and NIR) to emphasise the total reflectance and albedo effects. To ensure stability, the numerator and denominator components were averaged over the 12 months before the final calculation. The fourth and fifth datasets were derived from the PCA and applied to February and August. The PCA focused on maximising the variance between the spectral bands [64], with band 3 (Green) and band 4 (Red) making the largest contribution. The first principal component from February was used directly, while the values from August were inverted to ensure comparability. Finally, the sixth dataset, a difference image between the humid and arid Kennaugh datasets, emphasised contrasts, such as at sinkholes, where reflectance variations were more pronounced, facilitating the identification of features that respond to seasonal changes. These various pre-processing steps provided a solid basis for analysing spectral and temporal variations under different environmental conditions.

### 4.2. Multi-Scale Filter Approach for Surface Feature Detection

In the next step, a multi-scale filter approach based on specially designed filter kernels was applied to the pre-processed and edited data. The basic idea of recognising surface features (sinkholes and takyrs) is based on analysing the albedo values (total intensity in the satellite images) influenced by natural illumination. The depressions (sinkholes) lead to reduced reflectance values due to the shadow effect, so that they are recognisable as darker regions in the satellite data. Regarding the L2A product, terrain effects like shadows are corrected in principle. But, as the sinkholes are too small to be captured in the Copernicus DEM, the DEM-based image correction does not remove their shadows. In contrast to sinkholes, the takyrs—flat and sandy formations that are widespread in Kazakhstan—are, due to their flatness, not affected by shadow effects and have a higher reflectance, making them appear brighter in the images. In comparison, sinkholes are characterised by a dark centre and bright edges, whereas takyrs show the inverse spatial signature: bright centre and dark edges. In addition, the approach takes advantage of the low or absent vegetation and lack of urban development in the region, which would otherwise obstruct the line of sight and necessarily influence the measured albedo values.

The distinction between highly reflective takyrs and weakly reflective sinkholes serves as the basis for the identification of surface features with the multi-scale filters. The Laplace-Gaussian filter (LoG), second derivative of the Gaussian function, is used as a model for the design of the multi-scale filter masks, which is an important image processing operator. It is used to recognise local intensity peaks, edges, and circular features that stand out strongly from their surroundings.

**Figure 3:**
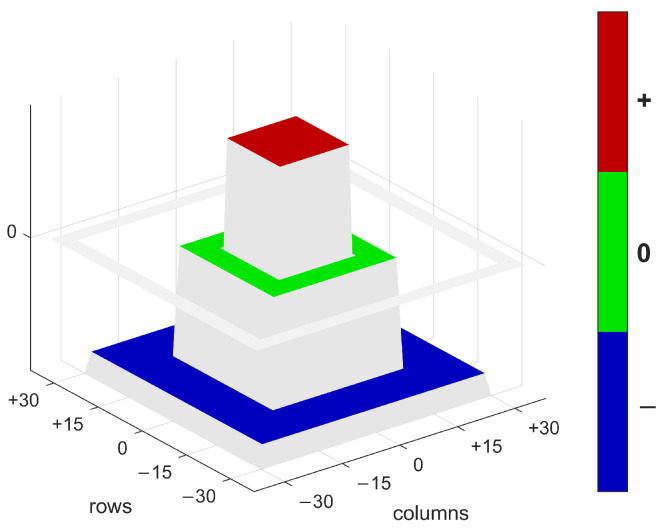
3D representation of the filter mask for sinkhole detection. The different colours represent the inner, middle, and outer masks with the corresponding values, which give different weighting to an approximation of the three areas of the sinkhole.

It combines two mathematical processes: Gaussian smoothing, which reduces noise and minor deviations, and the Laplacian operator, which emphasises regions with strong curvature, i.e., rapid intensity changes to both sides of the envisaged location. Thus, the filter emphasises the differences between a central point and its surroundings and can detect both dark and bright regions based on the absolute value of the filter response. As the LoG filter is very computationally intensive, an approximation using box filtering is applied, which is implemented as a multi-scale filter bank in order to appropriately represent the different sizes of the sinkholes. The approximation is based on a multistage procedure so that three filter masks of different sizes (the outer, centre, and inner mask) are linked together (Figure 3). In the context of detection, these three stages correspond, respectively, to the surrounding surface (green ring), the transition zone (inner blue ring), and the sinkhole shadow (red square). While the transition between positive and negative values is typically just a line, it is explicitly widened to a zone with no influence on the filtered image. This design allows the shadow to deviate from the predefined circular shape without changing the value of the convolutional layer. The different side lengths of the boxes are enlarged iteratively, resulting in a filter bank of 23 kernels.

The smallest detectable sinkhole size is at least 10 m during the iterative filtering; the mask sets, each with three scales, were applied to the six input datasets in MATLAB R2021b, generating filtered images for each scale. The result for a given scale was calculated using the following Equation (1), where the outer, middle, and inner box variables already contain the filtered image according to Figure 3.

(1) LocalCurvature=Inner Box+Middle Box−Outer Box

This image processing method enabled the detection of sinkhole-like structures at different scales. After that, the filter responses were analysed using profile diagrams of the given reference sinkholes. These plots showed which filter scales best matched the size of the sinkhole, with peaks corresponding to sinkhole depressions. The thresholds for sinkhole detection were derived from the maxima in these profiles to ensure a clear separation between sinkholes and background noise. These thresholds are then converted to values larger/smaller than the corresponding value, with a class (sinkhole/takyr) assigned depending on whether the value is positive or negative. In order to validate the recognition results, statistical metrics such as the Jaccard similarity coefficient, completeness, and correctness were calculated using the vectorised data and the available reference data. For takyrs, the recognition success was evaluated based on the total number of formations identified.

### 4.3. Combined Doline Vegetation Index

The Combined Doline Vegetation Index (CDVI) was developed to address the specific challenge of detecting sinkholes in Kazakhstan’s arid and semi-arid regions, where overlapping spectral and structural characteristics of vegetation, bare surfaces, and takyr-like areas complicate conventional detection methods. Vegetation in these areas, e.g., grass or shrub-like features, in particular, may also be small-scale, and existing EO-based datasets may not suffice due to their too-high spatial resolution. Furthermore, the vegetation there may even appear roundish, potentially leading traditional filtering approaches to confuse these vegetative features with sinkholes.

To overcome these challenges, the CDVI employs a mixed-temporal (multi-season), multi-sensor approach that leverages the complementary strengths of ESA’s Sentinel-1 SAR and Sentinel-2 optical data, acquired in different seasons. This approach capitalises on the unique capabilities of each sensor to address both spectral and structural complexities while accounting for seasonal variability [65]. Arid season data, such as Sentinel-1 SAR from 6 August 2023, emphasise structural contrasts in sinkholes due to sparse vegetation and low soil moisture, enabling the detection of bare sinkhole surfaces through unique scattering patterns. In contrast, humid-season data, such as Sentinel-2 observations from 30 March 2023, provide critical multispectral information, particularly in the near-infrared (NIR) range, highlighting vegetation dynamics and soil moisture. This integration ensures that the spectral and structural variations critical to sinkhole detection are captured comprehensively, addressing the limitations of single-sensor methods.

The fusion of Sentinel-1 SAR and Sentinel-2 optical data (B2, B3, B4, B8) leverages a unified radiometric framework termed Hypercomplex Bases (HCB) [63]. In this methodology, Sentinel-2 reflectance values are initially normalised to balance the spectral channels by reducing the influence of the NIR band. These normalised values are then converted into Kennaugh-like elements via linear combinations [66], resulting in one total reflectance element and three spectral elements. Sentinel-1 provides VV- and VH-polarised SAR imagery in the C-band, which is sensitive to structures approximately 5 cm in size. VV co-polarisation typically exhibits the strongest backscatter over terrestrial surfaces, whereas VH cross-polarisation is primarily influenced by volume scattering effects, such as those observed in areas with dense vegetation. The complex SAR images undergo preprocessing based on the framework provided in [67], which is based on the Multi-SAR processor [68]. This process calculates four Kennaugh elements ($k_{0}$, $k_{1}$, $k_{5}$, and $k_{8}$), preserving the complete polarimetric information [62]. These elements, representing intensities and intensity differences, were geocoded to the respective geographic zone. Final normalisation ensures consistent data ranges and allows the efficient storage of UInt16 digital numbers, analogous to the Sentinel-2 data. The datasets are subsequently fused using the linear HCB approach, producing a fused and normalised dataset consisting of one total intensity element ($k_{0}$) and seven spectral/polarimetric elements ($k_{1}-k_{7}$), as detailed in [63] and applied in [69]. The development of the CDVI was grounded by statistical analyses, including T-tests and ANOVA, which guided the selection of *k* bands and their weighted combinations in the index Equation (2). Correlation analyses further optimised the methodology, ensuring the high separability of critical land cover types such as sinkholes, vegetative features, and takyr surfaces. The CDVI is ultimately computed as a weighted combination of Kennaugh elements derived from the fused dataset:

(2) $$\mathrm{CDVI}=\frac{k_{5}+k_{7}}{2}-k_{1}+\frac{k_{0}+k_{6}+k_{7}-k_{1}-k_{2}}{3}$$

Each component of Equation (2) contributes to the enhanced separability of sinkholes and vegetative features from the other main land cover types (see Table 3).

### 4.4. Decision Criteria and Implementation Within GIS Framework

The complexity of the study area in Kazakhstan means that no uniform value range can be identified after applying the multi-scale filter. Instead of one uniform threshold, the threshold is, thus, adapted to each dataset (Table 2). Two asymmetric thresholds define significant deviations from zero, one for sinkholes (characterised by a typical dark centre under non-vegetated conditions, though this may vary due to vegetation presence) and another for takyrs (bright centre). In addition, the developed CDVI is integrated as a further decision criterion in order to minimise the disturbing influences of the existing low vegetation and, therefore, to support the detection results obtained. Vegetation next to takyrs could falsely be recognised as a sinkhole due to shadow effects in contrast to the bright response of the takyr centre. For this reason, a buffer zone around the takyrs is calculated to identify the vegetation polygons that are not sinkholes. These polygons are removed from the detection layers.

**Figure 4:**
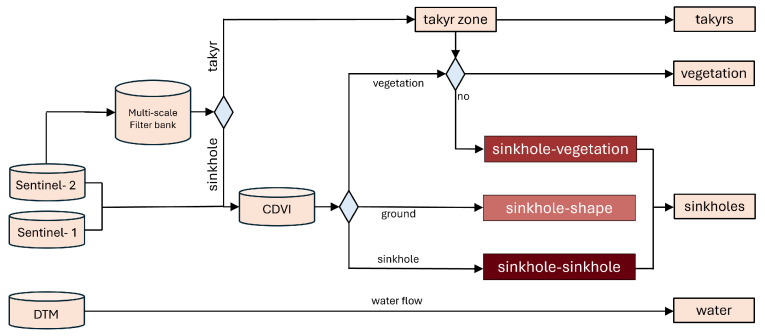
Visualisation of the whole workflow: Sentinel-1 and Sentinel-2 data serve as input to the CDVI and for the multi-scale filter bank. A sophisticated decision tree classification leads to polygons of takyrs, vegetation, and sinkholes. Flow accumulation of the DTM delivers the surface water runoff.

Concerning the support of the detection results, the next step is a rule-based classification into three categories, which is visualised in Figure 4. This is based on the combination of two layers, the CDVI layers with three categories (sinkholes, vegetation, and ground) and the multi-scale analysis with detected sinkholes and takyr. The aim is to create a final grid for each individual dataset that classifies the sinkholes into different categories.

The final goal is to create a merged dataset from the individual datasets that can be used as a reference basis. Only the individual datasets that perform successfully in the respective validation with the reference data should be used here. These individual datasets are then merged and, after a comprehensive validation with metrics such as f1-score, sensitivity, and precision, the quality of the final result is assessed in comparison to the individual datasets. It also analyses which data pre-processing method is best suited for detection in order to achieve a robust and reliable result.

### 4.5. Geospatial Distribution

Finally, in order to obtain a comprehensive overview of the relationships between sinkholes, takyr, vegetation, and hydrology in the study area, a spatial analysis is carried out to visualise and understand their distribution and interactions. Hereby, the sinkhole classifications are used with no further differentiation. Gaussian filtering (sinkholes, takyrs, and vegetation with a sigma of 1 km, for water 500 m) is used to create heat maps, which are merged into a visualisation, with each land class assigned its own colour (e.g., red for sinkholes, blue for water, green for vegetation). Overlapping regions in the composite visualisation highlight areas of interaction between land classes. To further explore these relationships, hex-bin plots are created to visualise the density of co-occurrence of land classes. These plots use colour and hexagonal bins to indicate the concentration of data points, while additional histograms for each class show the individual data distributions. Filtering based on percentiles excludes extreme outliers, focusses the analysis on trends, and avoids distortions caused by rare anomalies. This approach enables a detailed spatial understanding of soil classes and reveals their relationships, potential correlations, and underlying patterns, providing valuable insights for subsequent geospatial and ecological studies.

## 5. Results

The procedural workflow leads to various results; an initial intermediate result is produced after the image processing procedure. This is then blended with the CDVI and visualised using different decision criteria. Furthermore, metric analyses are carried out to validate the detection results. Finally, the different land classes are linked to each other in order to carry out a spatial analysis, which should provide conclusions about possible correlations.

### 5.1. Sinkhole Detection by Multi-Scale Filtering

**Figure 5:**
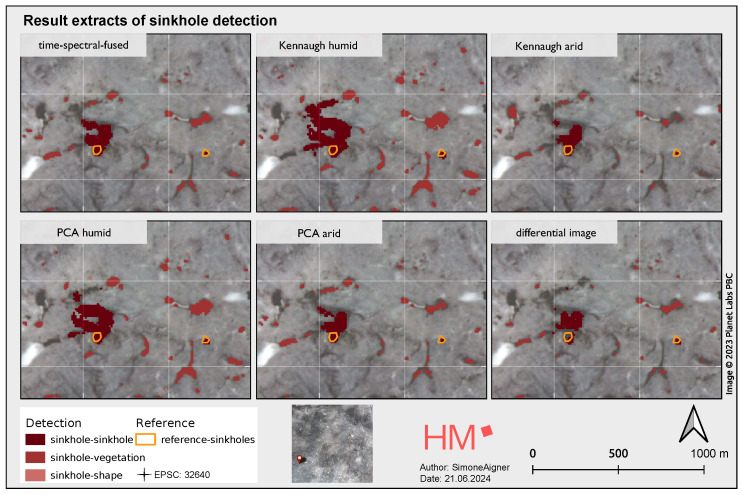
Result of the sinkhole detection from the image processing workflow after application of the decision criteria and classification (sinkhole-sinkhole, sinkhole-vegetation, and sinkhole-shape), whereby the individual results are compared with the reference data.

Figure 5 shows the intersection of the sinkhole detections region from the image processing method with the CDVI, with the strength of the red tone indicating the type of sinkhole or detection. The sinkhole-sinkhole category indicates that a circular shape has been identified as a sinkhole by both the pattern-based detection method and the CDVI. Sinkhole vegetation represents a circular shape that was detected by the CDVI index and categorised as vegetation, while sinkhole shape refers only to pattern-based detection. In general, it can be seen that sinkholes were recognised evenly in all datasets compared to the reference dataset.

### 5.2. Sinkhole Verification by CDVI

**Figure 6:**
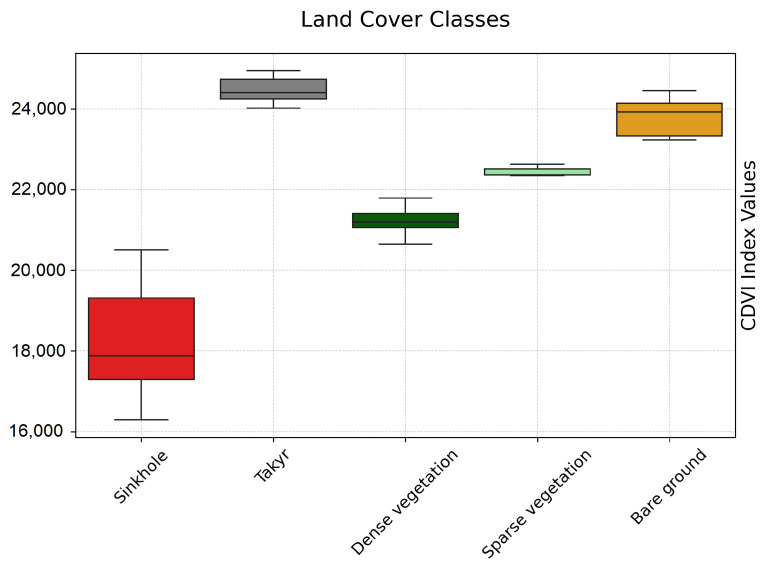
Comparison of CDVI values across the AOI’s dominant land cover classes. Boxplots illustrate the variability and central tendencies of CDVI values for sinkhole, takyr, dense vegetation, sparse vegetation, and bare ground classes, highlighting distinct ranges.

The CDVI was rigorously validated using multiple reference datasets to ensure its robustness and applicability across varied land cover types, including sinkholes, takyr surfaces, and vegetation. These reference datasets provided a solid foundation for assessing the CDVI’s performance in distinguishing key features and land cover types in arid and semi-arid environments. The CDVI’s capability to distinguish sinkholes and other land cover classes is depicted in Figure 6. Boxplots illustrate the variability and central tendencies of CDVI values for dominant land cover types, including sinkholes, takyr surfaces, dense vegetation, sparse vegetation, and bare ground. The distinct ranges highlighted in the boxplots demonstrate the CDVI’s robustness in reducing misclassification and ensuring clear separability between these classes.

**Figure 7:**
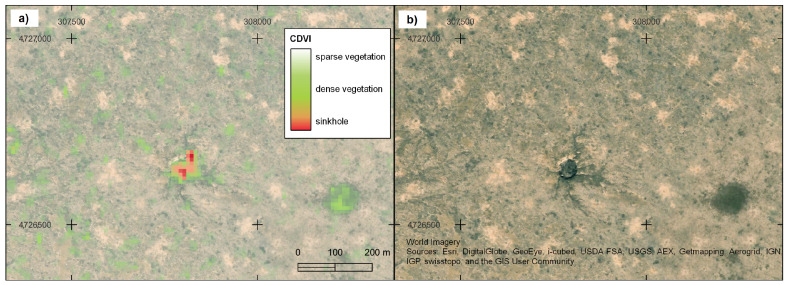
Spatial visualisation of CDVI performance: CDVI values overlaid on World Imagery [60], illustrating vegetation patterns and CDVI correspondence; (**a**) zoomed-in view of a sinkhole with sparse vegetation and CDVI values; and (**b**) the same sinkhole area as in (**a**), showing only World Imagery [60] for direct visual comparison.

The spatial visualisation in Figure 7 further highlights the CDVI’s effectiveness in delineating sinkholes and vegetation patterns: (a) zoomed-in view of a sinkhole with sparse vegetation and its corresponding CDVI values overlaid on WorldImagery [60], showing a clear correspondence between vegetation patterns and CDVI values; and (b) the same sinkhole area without the CDVI overlay, providing a direct comparison to raw imagery. These visualisations underscore the CDVI’s capacity to accurately map sinkholes and surrounding vegetation, offering practical insights into its spatial applicability.

**Figure 8:**
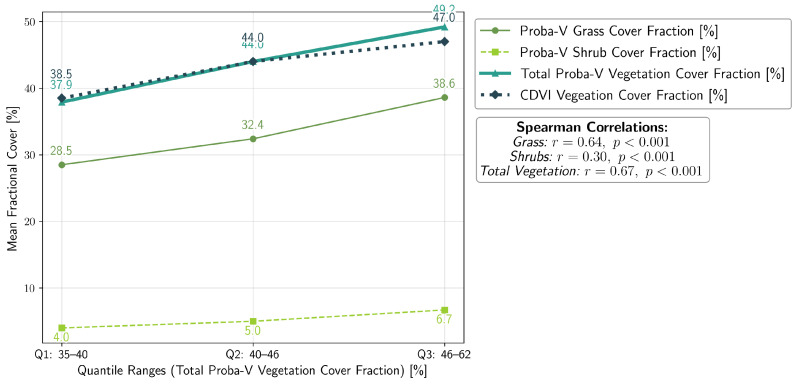
Comparison of CDVI Fractional Vegetation Cover (%) with Proba-V LC100 2019 [61] Grass, Shrub, and Total Vegetation Cover Fractions (%) across Total Vegetation quantiles (Q1–Q3). Spearman correlation coefficients for each dataset are included.

To validate the performance of the CDVI in detecting vegetation presence, its results were compared with the Proba-V LC100 global land cover product from 2019 [61]. The Proba-V dataset, part of the Copernicus Global Land Service, provides fractional grass and shrub cover values (0–100%) at a spatial resolution of approximately 100 m. While Proba-V is tailored to estimate grass and shrub coverage, the CDVI was designed to broadly detect vegetation presence, including grasses and small greenery, in arid and semi-arid landscapes such as southern Kazakhstan’s Mangystau region. A binary vegetation mask was derived from the CDVI using a threshold determined in Figure 6. This classified vegetation presence as 1 (vegetation) and absence as 0 (no vegetation). To facilitate direct comparison with Proba-V, the CDVI raster was reprojected and aligned to the Proba-V grid (100 m resolution). Using a summation resampling method, the number of 10×10m vegetation pixels (1) within each Proba-V cell was counted. The fractional vegetation cover was then calculated for each Proba-V cell by dividing the vegetation pixel count by the total number of valid pixels in the cell. To smooth spatial variations and reduce noise, a Gaussian filter (σ=10) was applied to the CDVI fractional vegetation cover data. This step produced a continuous raster surface comparable to Proba-V’s coarser resolution datasets. A Total Proba-V Vegetation Cover Fraction raster was calculated by summing the Proba-V Grass and Shrub Cover Fractions at each pixel. Values exceeding 100% were capped at 100%, and pixels marked as NoData in either layer were excluded from the calculation. This raster provides a combined measure of vegetation presence, serving as a reference for validating CDVI results. The validation was conducted using quantile-based analysis. Proba-V Total Vegetation Cover Fractions were divided into quantiles based on the 25th, 50th, and 75th percentiles, representing low, medium, and high vegetation cover (Quantiles Q1–Q3). CDVI results were then compared to Proba-V datasets (Grass, Shrubs, and Total Vegetation). Mean cover fractions, ranges, and quantile sizes for each dataset were computed. Spearman’s correlation coefficients (*r*) were calculated to quantify the relationship between CDVI and Proba-V datasets (Grass, Shrubs, and Total Vegetation). Strong correlations were observed, with the highest correlation between CDVI and Total Vegetation (r=0.67,p<0.001). Figure 8 compares the mean fractional vegetation cover derived from CDVI and Proba-V [61] datasets across Total Vegetation quantiles (Q1–Q3). The results reveal a close alignment between CDVI and Proba-V Total Vegetation, with a slightly weaker correlation for Proba-V Grass and Shrubs. Numerical annotations highlight the mean values for each dataset within each quantile.

**Figure 9:**
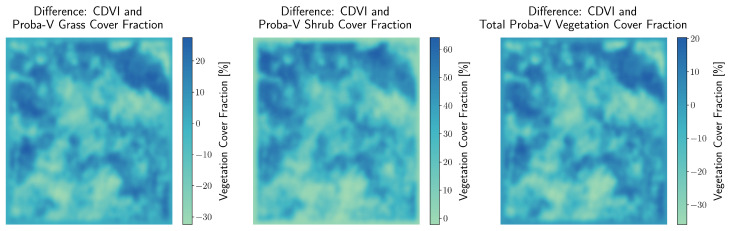
Spatial differences between CDVI Fractional Vegetation Cover (%) and Proba-V LC100 [61] Grass, Shrub, and Total Vegetation Cover Fractions (%). Green hues represent areas where Proba-V values are higher, while brown hues indicate higher CDVI values.

Figure 9 presents spatial difference maps, visualising the discrepancy between smoothed CDVI fractional vegetation cover and each Proba-V [61] dataset (Grass, Shrubs, and Total Vegetation). These maps reveal localised mismatches in vegetation cover, which may reflect CDVI’s finer resolution and broader vegetation detection capabilities.

The statistics for Total Vegetation quantiles, shown in Table 4, highlight the alignment between CDVI and the calculated Proba-V based [61] Total Vegetation data. CDVI provides detailed fractional vegetation cover estimates, capturing finer variations within each quantile.

The CDVI demonstrates strong agreement with Proba-V [61] datasets, particularly Total Vegetation (r=0.67), confirming its ability to effectively detect vegetation presence in arid and semi-arid landscapes. The weaker correlation with Shrubs (r=0.30) suggests that the CDVI is less tailored to detect sparse woody vegetation. These results highlight the complementary nature of the CDVI and Proba-V [61] datasets, with CDVI excelling in capturing smaller-scale vegetation patterns, such as interspersed grasses.

**Figure 10:**
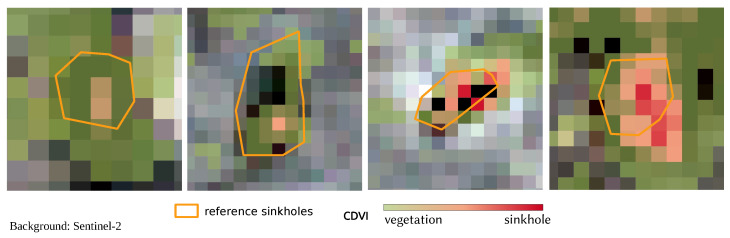
CDVI overlay on Sentinel-2 (©ESA 2023) imagery, showing vegetation (green) and sinkholes (red) on a continuous colour scale. GPS-based sinkhole references [49] are shown in orange as vector overlays. Variability in sinkhole characteristics, such as brighter centres, can result from vegetation growth within sinkhole depressions, highlighting the importance of the CDVI in distinguishing sinkholes from vegetative features.

The practical utility of the CDVI is illustrated in Figure 10, where the index is overlaid on Sentinel-2 imagery. Vegetation is represented in green, sinkholes in red, and known sinkholes [49] in orange. This example underscores the CDVI’s role as a plausibility control tool for vegetation and sinkhole mapping.

The CDVI’s fine resolution and multi-seasonal design make it an effective tool for ecological and geomorphological research, enabling the precise mapping of vegetation patterns in challenging environments, such as sparse grasses mixed with bare ground, which are characteristic of southern Kazakhstan’s landscape [53,54]—and serving as a supplementary reference for sinkhole detection in this study.

### 5.3. Accuracy of Verified Sinkhole Map

The accuracy analysis is presented in Table 5 and Table 6, which provide a detailed comparison of the metrics for each dataset with the reference dataset created for both the sinkholes and the takyrs. It can be seen that the individual differential image dataset was not used for the merged final dataset. This is because it achieved less than 49% when comparing the completeness with the reference data, whereas the other datasets achieved up to 76%.

In Table 5, focusing on sinkholes, the PCA arid dataset shows the highest performance with an f1-score of 83%, a sensitivity of 79%, and a precision of 88%. The temporal-spectral dataset also shows similar results with an f1-score of 82% and equal values for sensitivity and precision (82%). Table 6 shows the metrics for the takyrs detection. The PCA-arid dataset also stands out here with the highest f1-score of 0.96, a sensitivity of 93%, and a precision of 99%. The temporal-spectral dataset follows with an f1-score of 85%, with a sensitivity and precision of 96% and 76%, respectively. The Kennaugh arid dataset, on the other hand, has a lower precision (47%) despite a high sensitivity of 97%.

### 5.4. Geospatial Distribution

**Figure 11:**
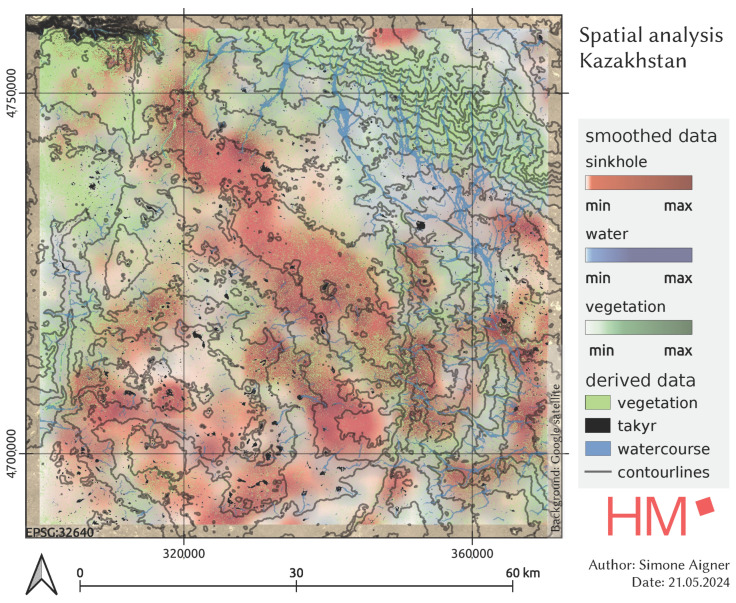
Spatial distribution of land cover classes in the test area of Kazakhstan, showcasing smoothed data for sinkholes (red), water (blue), and vegetation (green), alongside derived data for vegetation, takyr (black), watercourses, and contour lines (interval 10 m) from the digital terrain model.

The map in Figure 11 shows the spatial overlay of the different land cover classes in the test area in Kazakhstan. The black polygons are the recognised takyrs, which are very evenly distributed. The watercourses are displayed in two ways: as a smoothed raster layer and as vectorised lines for better clarity. With regard to the hydrology, it can be seen that the course of the river is interrupted in the area of the sinkholes, but that some sinkhole clusters are connected by the course of the river. The vegetation density is also visualised using CDVI. Contour lines derived from the digital terrain model (DEM) represent topographical features, with the denser spacing in the north-eastern section indicating a steep slope. The north-western section also has a steep slope leading to a ravine. In contrast, the terrain in the areas with many sinkholes is predominantly flat. This map is used to analyse the spatial relationships between sinkholes, takyrs, water, and vegetation in a region.

**Figure 12:**
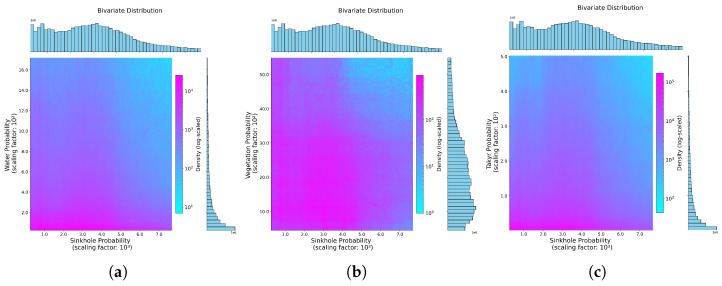
Hex-bin diagrams showing the bivariate distribution between sinkholes and water (**left**), sinkholes and vegetation (**centre**), and sinkholes and takyr (**right**) and illustrating the density distribution and correlations in the test area in Kazakhstan. (**a**) Sinkholes vs. water, (**b**) sinkholes vs. vegetation, and (**c**) sinkholes vs. takyr.

The hex-bin plots in Figure 12 were used for further analysis to investigate the relationships between sinkholes and water, vegetation, and takyrs. The hex-bin plot for sinkholes and water in Figure 12a shows the highest density of data points in areas where neither sinkholes nor water are present (low values for both classes). The plot also reveals a partial overlap of these classes, as indicated by a faint pink hue and the horizontal histogram for sinkholes, especially at mid-range sinkhole values closer to their centres. The plot for sinkholes and vegetation in Figure 12b highlights regions with a high density of low values for both classes, suggesting areas where neither sinkholes nor vegetation are prominent. However, overlaps are observed at mid-range sinkhole values, evidenced by colour variations and consistent histograms for both variables. The sinkhole and takyr plot in Figure 12c demonstrates a predominant density of low values for both classes, indicating minimal co-occurrence. Nevertheless, regions with diverse sinkhole and takyr values are apparent. The histogram reveals that takyr values are predominantly low, while sinkhole values have a broader distribution, including higher ranges.

## 6. Discussion

The discussion now critically assesses the results derived above. The new findings will be examined from various aspects: the workflow itself, possible simplifications and enhancements in future studies, the transferability of the method, existing limitations, and the method’s application for change analysis. The focus generally lies on semi-arid regions with sparse vegetation and an absence of exhaustive high-resolution geodata. Finally, the lessons learned are summarised and answers to the research hypotheses are given.

### 6.1. Workflow Design

The workflow consists of a multi-scale pattern recognition based on the albedo, i.e., just one information layer. Thanks to the filter design based on a multi-scale bank of boxcar filters, the calculation is simple and extremely fast. Therefore, it is easily applicable to large coverages. Side effects of using a the discrete filter mask instead of a more-or-less continuous LoG-filter did not show up in the results. The multi-scale curvature filter highlights locations with a high local curvature in different scales. The sign of the curvature allows for the discrimination of sinkholes (dark in the middle and bright at the edge) from takyrs (bright in the middle and dark at the edges). One emerging problem is the confusion of sinkholes with vegetation next to takyrs. As takyrs represent sinks for surface water, they are typically surrounded by vegetation, which produces a spatial signature similar to the signature of sinkholes, as the vegetation appears dark in front of a bright background. Therefore, a plausibility check by the CDVI was implemented. This multi-temporal fusion of radar and optical data from rainy and arid seasons delivers a clear distinction between vegetation and sinkholes. The combination of multi-scale filter banks and CDVI consequently leads to a self-controlling approach for the mapping of sinkholes, takyrs, and vegetation with minimal computational effort.

### 6.2. Simplifications

Several combinations of monthly Sentinel-2 acquisitions over one year were evaluated. The comprehensive study found that the fused dataset over one year is not superior to the evaluation of single, cloud-free acquisitions of the arid period decomposed via principal component analysis (see Table 5 and Table 6). The lower performance of the ’Kennaugh arid’ dataset compared to PCA arises from differences in how the two approaches handle variability in arid conditions. The Kennaugh dataset, dependent on spectral and structural contrasts, is less effective in arid environments where such contrasts are subdued. PCA, by isolating components with the highest variance, excels in extracting subtle distinctions between geomorphological features and their surroundings, even under conditions of low overall variability. As the greatest variation can be found between the rainy and arid seasons, a second Sentinel-2 acquisition at the end of the rainy season, when the landscape is still humid and vegetation is alive, but clouds are rare, is useful as input to the CDVI. One additional Sentinel-1 scene during the arid season is sufficient. Thus, only three Sentinel images per year already allow for the delineation of sinkholes in Kazahkstan. Preliminary studies that simulated the shadowing to the varying incidence angle of the sun in summer (steep) and winter (flat) showed that its influence is negligible. Thinking of shadowing, a combination of the albedo from Sentinel-2 and the intensity of Sentinel-1 is most promising. Due to the different acquisition geometry (range projection, in the case of Sentinel-1) and illumination (active vs. passive), the two Sentinels deliver different shadows for the same sinkhole, which are expected to stabilise the detection. The high potential of Sentinel-1 already showed up in the CDVI. Regarding the processing level, L1C data of Sentinel-2 are expected to be sufficient because the image fusion over time is no longer envisaged. With respect to Sentinel-1, the CDVI still contains the full polarimetric information of the Kennaugh decomposition. Future studies may evaluate the information loss by reducing the intensity values in VV and VH with phase information. In this case, the use of the much smaller GRD products, plus geocoding and gamma calibration, could further accelerate the processing.

### 6.3. Enhancements

The multi-scale approach combines responses from different scales in one layer. The study proved that this is enough to reliably detect sinkholes and takyrs. As both geological features are roundish and produce an ellipse-like shadow, one could imagine filtering with multi-scale directional filter banks [70,71]. At this point, it has to be stated that the use of directional filter banks also causes a much higher computational effort than that of an undirected multi-scale approach [62,70]. Recent studies build upon box-like, undirected filters, but decorrelate the image pyramid levels by removing large-scale structures before refining the scale. They estimate local ellipses from one smoothing, two gradient, and three curvature filters [72,73], i.e., only six filter masks per scale instead of several tens in the case of Schmittlets [70]. Hence, the computational effort is manageable. Furthermore, the approach directly delivers the location, extent, and orientation of the ellipses as parameters without the need to vectorise an image output, which is, again, time-saving. The proposed methodology would also enable time series analysis [73], which will be discussed separately. One drawback of the current implementation is the required definition of fixed thresholds for the detection, which leads to a relatively complex decision tree when combining the single results (Figure 4). This threshold selection could possibly be automated or replaced by the above-mentioned ellipse approach.

### 6.4. Limitations

The presented methodology also has clear limitations. As our approach uses a natural illumination by the sun and an artificial illumination by a SAR sensor, upright structures like vegetation and buildings impede the view to sinkholes. Additionally, they produce their own shadows that cannot be distinguished from shadows caused by terrain features. On one hand, buildings could simply be excluded using OpenStreetMap data, if the dataset was complete enough. But, sinkholes within settlements cannot be captured by this method. The same applies to sinkholes within dense vegetation, like forests. Therefore, the presented method is restricted to semi-arid to arid environments with only sparse vegetation and few settlements. Another critical landscape is mountains. Although it is possible, in principle, to estimate shadows from strong topography in the image by the help of the incidence angle of the illumination and imaging geometry, there might be confusion around the shadow areas. Furthermore, the shadow areas cannot be mapped, i.e., they stay empty and a data fusion from multiple sources is indispensable. To conclude, a relatively flat topography, as apparent in the studied area, simplifies the interpretation.

Also, while the proposed method achieves high accuracy and demonstrates significant potential for scalable sinkhole detection in data-scarce regions, a direct comparison with existing methods was not feasible due to the lack of high-resolution ground truth data in the study area. However, a related study conducted in Bavaria, Germany, using high-resolution Digital Terrain Models and official reference datasets, achieved a detection accuracy of 75% [74]. This demonstrates the robustness and flexibility of the methodology across diverse environmental and data contexts. Future research could focus on applying this methodology to regions with established datasets from conventional techniques, enabling a quantitative assessment of its relative performance. This would further validate the progressiveness of the approach and refine its applicability across diverse environments.

### 6.5. Transferability

As the methodology is restricted to the use of only two Sentinel-1 and Sentinel-2 images, it is transferable to any place where these two datasets are available. This enables a global coverage. Nevertheless, one has to respect the limitations formulated above: The methodology is applicable, but not all sinkholes will be detected, and confusion with other upright structures will possibly occur in vegetated or settled landscapes. Regarding other sensors, the method can also be applied to any optical sensor that measures the albedo, i.e., also the the PAN channel of Landsat (15 m pixels) or the total reflectance of Planet Scope (3.7 m pixels). The same goes for the SAR part. Sentinel-1 could possibly be replaced by TerraSAR-X, providing higher spatial resolution and varying incidence angles. It is important to mention that the use of other sensors (except for Landsat) is always linked to fee-based acquisitions and, in some cases, to reduced spatial and temporal coverage. One promising strategy would be to identify focus regions with a high occurrence of sinkholes by the help of the proposed method and, then, to refine the spatial resolution of the sinkhole map especially in this area by using other sensors. This would, again, represent a multi-scale approach, but this time, by multi-sensor data fusion on a decision level [65].

### 6.6. Change Analysis

Thanks to the vast and long-term availability of Sentinel data, it is feasible to repeat such a mapping of sinkholes and takyrs on a yearly basis. This is a major advance in comparison to the analysis of DEMs which are only measured once in a decade if at all. Two important aspects are evaluated when comparing multi-year maps. First, the stability of the algorithm could by proven. As only few reference data are available for our study site, the absolute accuracy assessment by ground truth might possibly be biased by the few sinkholes mapped in field campaigns. This problem of unsure ground truth accuracy cannot be solved without additional extensive and necessarily also expensive field campaigns. But, the precision (repeatability accuracy) of the sinkhole detection can be assessed by comparing the results from one year to those from another. One could statistically evaluate the locations and extents of the mapped sinkholes and takyrs. Assuming that most of the terrain features stay stable, the results should only show minor deviations. Secondly, if local deviations are observed, this might hint at a real change and, therefore, an instability of the terrain. This information is most critical with regard to infrastructure projects like Hyrasia One. Using Sentinel-1 and Sentinel-2 data, the time series could possible by being backdated to start in 2015 until now. That means that by next year, the study could fuse 10 maps of sinkholes and takyrs in order to stabilise the information and to highlight unstable areas.

### 6.7. Lessons Learned

Let us now respond to the three research hypotheses stated in the beginning of the article. (1) Natural illumination indeed enables the detection of terrain features like sinkholes, similar to the shading of digital elevation models. One restriction is that the terrain has to be relatively flat, without standing vegetation or buildings. This requirement is fulfilled for our study area. Some confusion with vegetation, mainly around takyrs, is noted and needs further examination. By the help of the newly developed CDVI, an integrated plausibility control is implemented that allows to remove the false positives. (2) Yes, it is possible to discriminate between sinkholes and similar morphological terrain features only by evaluating satellite images of Sentinel-1 and Sentinel-2. The final map contains sinkholes, takyrs, and vegetation in separate layers. (3) We could also illustrate and analyse that there is a clear negative spatial correlation between areas with a high occurrence of sinkholes and superficial water bodies. Valleys with river flows care for superficial water discharge. They are visible and do not pose any problem to infrastructure projects. Sinkholes, on the contrary, lead to a subterraneous water discharge and the increase in karst caves. They pose an immense problem because the terrain becomes unstable over shorter or longer time periods. The study could show that sinkholes predominantly appear on plateaus in parallel to superficial water flows (Figure 11), but with a certain spatial distance from the water flow. These areas do not contain superficial drainage channels according to the DEM-based flow accumulation model. Thus, the water discharge has to take place underground. This insight is, for sure, only valid for the studied region and might be different in other karst regions of the world, but it is an interesting starting point for future follow-up studies.

## 7. Conclusions

This article introduces a new approach for the area-wide mapping of superficial karst features by the help of open, high-resolution satellite data originating from the Copernicus program of the European Space Agency. The methodology unfolds as a two-step self-controlling concept with multi-scale feature detection and subsequent feature validation by the newly developed Combined Doline Vegetation Index (CDVI). The study demonstrates the strengths, limitations, and future potential of the developed method for recognising karst features such as sinkholes and takyrs in arid and semi-arid environments. It is practical for large-scale applications due to the simplicity and computational efficiency of the light and shadow analysis in combination with freely available satellite data. According to the experiments, only one Sentinel-1 (in the arid season) and two Sentinel-2 scenes (one in the arid season and one in the rainy season) per year are sufficient, so that a sinkhole and takyr assessment on a yearly basis becomes feasible. Moreover, the robustness of the approach is increased by a double confirmation process that reduces uncertainties and improves reliability. The integration of hydrological and topographical data provides deeper insights into spatial relationships and supports sophisticated interpretations of geophysical processes. While the method performs well in sparsely vegetated and flat regions, problems are expected in densely vegetated, urbanised, or mountainous areas. The spatial analysis clearly shapes regions with a higher occurrence of sinkholes, which are mainly located on plateaus along river valleys. A flow accumulation model derived from the Copernicus Digital Elevation Model approves that there are no terrain structures that might support a superficial water discharge. The comprehensive temporal coverage of Sentinel data since 2015 enables meaningful time series analyses and provides valuable opportunities to monitor stability and detect changes over time. Despite limited reference data and the lack of complete ground truth, the high accuracy of the results indicates the potential to identify significant trends through multi-year comparisons. The results demonstrate the reliability of the algorithm and its ability to recognise dynamic changes in karst landscapes, providing important insights for assessing terrain stability and potential risks. The findings emphasise the growing importance of monitoring karst structures in the context of climate change. Changing precipitation patterns, including prolonged droughts and increased precipitation, have a significant impact on sinkhole dynamics. Droughts exacerbate soil aridness and wind erosion and destabilise fragile karst systems, while heavy rainfall accelerates dissolution processes and further undermines soil stability. Continuous monitoring is essential to minimise risk, protect groundwater from contamination, and ensure sustainable land use and infrastructure planning. Early detection enables proactive measures that reduce the long-term environmental and economic impact.

## Figures and Tables

**Table 1:** Sentinel-2 (©ESA 2022/2023) cloud coverage statistics (October 2022–September 2023).

Month	Date	Cloud Coverage (%)	Date	Cloud Coverage (%)
October	1 October	0.00	21 October	28.66
November	10 November	3.99	25 November	0.06
December	30 November	0.39	10 December	9.85
January	9 January	26.96	19 January	0.32
February	13 February	10.49	24 January	3.71
March	20 March	10.49	30 March	0.00
April	29 April	0.05	24 May	3.43
May	19 May	29.25	29 May	20.51
June	3 June	20.67	13 June	0.00
July	3 July	0.00	28 July	0.00
August	2 August	8.11	22 August	17.72
September	1 September	0.00	11 September	23.41

**Table 2:** Overview of refined datasets for sinkhole and takyr detection.

Dataset	Description	Purpose
Temporal-Spectral Dataset	Average spectral and temporal information over 12 months.	Captures long-term stability of surface features.
Kennaugh Dataset (February)	Total intensity ($K_{0}$) from normalised spectral bands for humid conditions.	Highlights features under humid soil and vegetation cover.
Kennaugh Dataset (August)	Total intensity ($K_{0}$) from normalised spectral bands for arid conditions.	Emphasises features under arid soil and sparse vegetation.
PCA Dataset (February)	First principal component emphasising variance in spectral bands during humid conditions.	Maximises variance for identifying features during humid conditions.
PCA Dataset (August)	Inverted first principal component for arid conditions.	Ensures comparability with February PCA, focusing on arid conditions.
Difference Image (February–August)	Difference of humid and arid Kennaugh datasets.	Enhances seasonal contrasts to highlight features like sinkholes.

**Table 3:** Description of the CDVI formula components and their roles.

CDVI Formula Component	Fused *k* Element Components	Description
$\frac{k_{5}\;+\;k_{7}}{2}$	Combines $k_{5}$ (spectral and structural coherence) and $k_{7}$ (volumetric scattering).	Highlights sinkhole boundaries and vegetation overlays by integrating radar structural features and spectral information from optical bands (e.g., NIR dominance for vegetation vigor).
$-k_{1}$	Subtraction of $k_{1}$ (intensity differences in radar and spectral contrasts).	Reduces surface reflectance artifacts, emphasising volumetric and structural features critical for separating vegetation and sinkholes from other land cover types.
$\frac{k_{0}\;+\;k_{6}\;+\;k_{7}\;-\;k_{1}\;-\;k_{2}}{3}$	Combines $k_{0}$ (total intensity), $k_{6}$ (spectral contrasts), and $k_{7}$ (volumetric scattering) while subtracting $k_{1}$ and $k_{2}$ to reduce noise.	Balances spectral and structural features across seasons. Prioritises sinkhole-specific features and vegetation separability, enabling robust classification from mixed-sensor data.

**Table 4:** Total Proba-V [61] Vegetation Cover Fraction (Grass and Shrubs) and Corresponding CDVI Vegetation Cover Fraction Statistics.

Total Vegetation Quantiles (%)	Quantile Sizes (%)	Proba-V Total Mean (%)	Proba-V Total Range (%)	CDVI Mean (%)	CDVI Range (%)
35–40	22.01	37.89	36–40	28.51	2.6–59.3
40–46	28.00	44.01	41–46	38.55	6.6–63.5
46–62	21.66	49.21	47–62	47.04	16.1–68.5

**Table 5:** Performance metrics (f1-score, sensitivity, and accuracy) for sinkhole detection in the merged reference dataset compared to the different individual datasets: temporal-spectral, Kennaugh (humid and arid), and PCA (humid and arid).

Dataset	f1-Score [%]	Sensitivity [%]	Precision [%]
temporal-spectral	82	82	82
Kennaugh humid	41	27	90
Kennaugh arid	52	75	40
PCA humid	39	25	87
PCA arid	83	79	88

**Table 6:** Performance metrics (f1-score, sensitivity, and accuracy) for takyr detection in the merged reference dataset compared to the different individual datasets: temporal-spectral, Kennaugh (humid and arid), and PCA (humid and arid).

Dataset	f1-Score [%]	Sensitivity [%]	Precision [%]
temporal-spectral	85	96	76
Kennaugh humid	78	64	99
Kennaugh arid	63	97	47
PCA humid	68	53	96
PCA arid	96	93	99

## Data Availability

Remote sensing data derived from public domain resources. Reference data were obtained from Svevind Energy Group.
